# Time course analyses of structural changes in the infrapatellar fat pad and synovial membrane during inflammation-induced persistent pain development in rat knee joint

**DOI:** 10.1186/s12891-018-2391-1

**Published:** 2019-01-05

**Authors:** Kei Inomata, Kunikazu Tsuji, Hiroaki Onuma, Takashi Hoshino, Mio Udo, Masako Akiyama, Yusuke Nakagawa, Hiroki Katagiri, Kazumasa Miyatake, Ichiro Sekiya, Takeshi Muneta, Hideyuki Koga

**Affiliations:** 1 Department of Joint Surgery and Sports Medicine, Graduate School, Tokyo Medical and Dental University, Tokyo, Japan; 2 Department of Cartilage Regeneration, Tokyo Medical and Dental University, 1-5-45, Yushima, Bunkyo-ku, Tokyo, 113-8510 Japan; 3 Research Administration Unit, Tokyo Medical and Dental University, Tokyo, Japan; 4 Center for Stem Cell and Regenerative Medicine, Tokyo Medical and Dental University, Tokyo, Japan; 5 National Hospital Organization Disaster Medical Center, Tokyo, Japan

## Abstract

**Background:**

Osteoarthritis (OA) is a common joint disease in aging societies, which is accompanied by chronic inflammation and degeneration of the joint structure. Inflammation of the infrapatellar fat pad (IFP) and synovial membrane (IFP surface) plays essential roles in persistent pain development in patients with OA. To identify the point during the inflammatory process critical for persistent pain development, we performed a time course histological analysis in a rat arthritis model.

**Methods:**

Wistar rats received single intra-articular injection of monoiodoacetic acid (MIA, 0.2 or 1.0 mg/30 μL) in the right knees or phosphate-buffered saline (PBS, 30 μL) as a control in the left knees. Pain avoidance behaviors (weight-bearing asymmetry and tactile hypersensitivity of the plantar surface of the hind paw) were evaluated on days 0, 1, 3, 5, 7, and 14 after injection. Histological assessments of the knee joint were performed on days 0, 1, 3, 5, and 7 after MIA injection.

**Results:**

Weight-bearing asymmetry was observed along with the onset of acute inflammation in both the low- (0.2 mg) and high-dose (1.0 mg) groups. In the low-dose group, weight-bearing asymmetry was completely reversed on day 10, indicating that joint pain seemed to alleviate between days 7 and 10. In contrast, we observed persistent joint pain after day 10 in the high-dose group. Histological assessments of the high-dose group indicated that the initial sign of inflammatory responses was observed in the perivascular region inside the IFP. Inflammatory cell infiltration from the perivascular region to the parenchymal region of the IFP was observed on day 3 and reached the IFP surface (synovial membrane) on day 7. Extensive fibrosis throughout the IFP was observed between days 5 and 7 after MIA injection.

**Conclusion:**

Our data indicated that acute joint pain occurs along with the onset of acute inflammatory process. Irreversible structural changes in the IFP, such as extensive fibrosis, are observed prior to persistent pain development. Thus, we consider that this process may play important roles in persistent pain development.

**Electronic supplementary material:**

The online version of this article (10.1186/s12891-018-2391-1) contains supplementary material, which is available to authorized users.

## Background

Osteoarthritis (OA) is one of the most common diseases in aging societies, which is accompanied by chronic joint inflammation, articular cartilage degeneration, and osteophyte formation [1, 2]. Epidemiological analyses in Japan suggested that the number of patients with radiographically identifiable knee OA (Kellgren-Lawrence score ≥ 2) was almost 25 million, which is approximately one-fifth of the Japanese population, and almost 30% of them (8 million) have symptoms that affect activities of daily living [3].

The major complaint of patients with knee OA is persistent pain, which significantly decreases both, the patients’ activities of daily living (ADL) and quality of life (QOL). Therefore, most of the current conservative treatment strategies for knee OA are based on symptom management by anti-inflammatory analgesics and improvement of joint mobility and flexibility by programed exercise (land or water based), weight control, and education [4]. However, some patients develop uncontrolled persistent knee pain with disease progression. In these cases, patients are pressed to make decisions for alternative treatments, such as total knee arthroplasty.

Mechanisms of persistent pain development in knee OA have largely not been elucidated. During inflammation or tissue injury, nociceptive pathways are activated by various mediators that sensitize primary afferent nerves in the joint (peripheral sensitization). Over time, these enhanced neuronal activities variegate the plasticity of second-order neurons in the central nervous system, making them more responsive to the signals from the periphery. Another important component in the process of persistent pain development is the structural changes in the joint tissues. Previous studies have reported that synovial fibrosis contributes to joint pain and stiffness [5–7]. However, it is not clear whether the structural changes of infrapatellar fat pad (IFP) and synovial membrane play important roles in persistent joint pain and to what degree. To answer this question, we performed time course histological assessments in the monoiodoacetic acid (MIA)-induced rat joint inflammation model.

Intra-articular injection of MIA is a well-characterized animal model for inflammation-induced OA [8–11]. By using this model, we have reported two different inflammation-induced articular cartilage degeneration models in rats [12, 13]. One is the low-dose model (0.2 mg), in which acute inflammation was observed within 3 days, peaked at 5 days, then alleviated after 7 days [12, 13]. In this model, we observed slow progression of articular cartilage degeneration after 28 days without apparent synovial inflammation after 14 days [12, 13]. The other is the high-dose (1.0 mg) model, in which the onset of acute inflammation was comparable with that of the low-dose model; however, obvious irreversible structural changes in the synovial membrane and IFP were observed after 5 days [12, 13]. We have compared the pain avoidance behavior between these two models and found that acute joint pain was observed along with the onset of the acute inflammatory responses in both experimental conditions. In the low-dose model, joint pain was rapidly alleviated after 7 days along with the resolution of acute inflammation [13]. In contrast, we noted the development of persistent pain in the high-dose model after 7 days [13]. We considered that these observations strongly suggested the importance of irreversible structural changes in the synovial membrane and IFP between 5 to 7 days during persistent pain development.

Based on this hypothesis, we performed time course histological analyses in the early stage after joint inflammation to elucidate which point during the irreversible structural changes in the synovial membrane and IFP is important for persistent pain development. Here, we report that extensive fibrotic changes occurred between days 5 and 7 after MIA injection.

## Methods

### Materials

MIA and paraformaldehyde were purchased from Sigma Aldrich (St. Louis, MO, USA). Isoflurane and ethylenediaminetetraacetic acid (EDTA) were purchased from Wako Pure Chemical Industries Ltd. (Osaka, Japan).

### Animals

Male Wistar rats (Charles River, Tokyo, Japan) at 8 weeks of age, 280–310 g in weight, were subjected to this study. The animals were housed under a 12 h/12 h light/dark cycle with food and water ad libitum. All procedures of this study were conducted in accordance with the protocols approved by the Institutional Animal Care and Use Committee (IACUC) of Tokyo Medical and Dental University.

### MIA injection

**Fig. 1:**
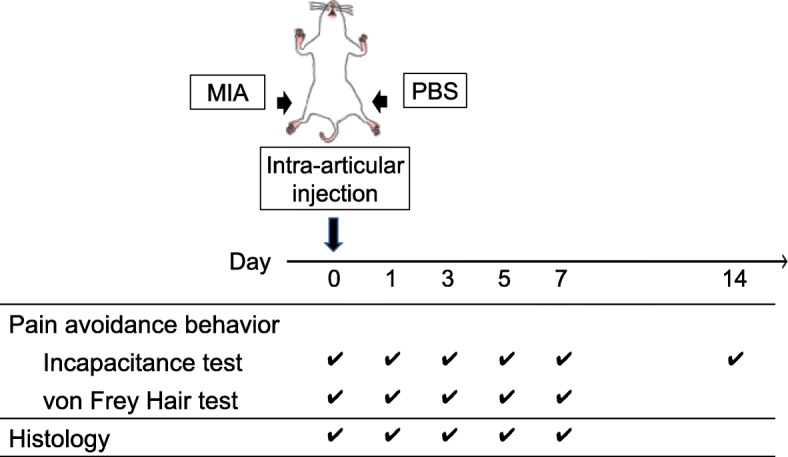
Study design. The right knee joint had an intra-articular injection of MIA at day 0. The left knee had PBS as a control. The pain avoidance behavior tests (Incapacitance test and von Frey Hair test) were performed as indicated. Histological evaluations (hematoxylin and eosin (HE), Masson Trichrome (MT)) and immunohistochemical assessments (anti-ED1 immuno-staining) were performed at days 0, 1, 3, 5, and 7. Steel’s test was employed to analyze the results of incapacitance test and von Frey Hair test. Asterisks (Fig. 2) indicated that the values were significantly altered with those of pre-experimental levels (0 day). Kruskal-Wallis test followed by Steel-Dwass test was performed to analyze the results of histological evaluations between 5 independent time points (Figs. 3, 4, and 5). Asterisks indicated that the values were significantly altered from those of pre-experimental levels (0 day)

Rats were anesthetized by inhalation of isoflurane (2% in oxygen, flow rate at 2 L/min). Under anesthesia, animals received single intra-articular injection of MIA (0.2 or 1.0 mg/30 μL in phosphate-buffered saline [PBS]) in the right knees to induce inflammation. As contralateral controls, 30 μL of PBS were injected into the left knees as described in our previous study (Fig. 1) [12]. To exclude the artificial effects of intra-articular injection (needlestick) in histological analyses for day 0 samples, animals received single intra-articular injection of PBS (30 μl) just before sacrifice. (day 0 sample, Fig. 1).

### Analysis of pain avoidance behavior

Severity of joint pain was quantitatively evaluated by weight-bearing asymmetry (incapacitance test, Linton Instrumentation, Norfolk, UK) and tactile hypersensitivity of the plantar surface of the hind paw (von Frey hair test, North Coast Medical, Inc., Gilroy, CA, USA) [9, 13–17]. These tests were performed to verify the reproducibility of the experimental model employed in this study with those of our previous report [13].

Weight-bearing asymmetry between the right (MIA side) and left (control side) limbs was measured on days 0, 1, 3, 5, 7, and 14 to reconfirm that pain persistence occurred after 7 days in the high-dose model (Fig. 1). Measurements were done over 100 times for each sample, and the percent weight of the MIA side (right hind limb) was calculated according to the equation below as described previously (*n* = 6) [13, 15].

$$ \left[\mathrm{Percent}\ \mathrm{of}\ \mathrm{weight}\ \mathrm{on}\ \mathrm{ipsilateral}\ \mathrm{limb}=\mathrm{weight}\ \mathrm{on}\ \mathrm{ipsilateral}\ \mathrm{limb}/\left(\mathrm{weight}\ \mathrm{on}\ \mathrm{ipsilateral}\ \mathrm{limb}+\mathrm{weight}\ \mathrm{on}\ \mathrm{contralateral}\ \mathrm{limb}\right)\times 100\right] $$

To verify the reproducible tactile hypersensitivity of the plantar surface of the hind paw was observed during the experimental period in this study, von Frey hair test was performed on days 0, 1, 3, 5, and 7 according to the methods described previously (n = 6, [13, 15]). In these experiments, the elasticity of the von Frey hairs was started from 300 g and gradually decreased until it reached 2 g. Maximum elasticity in which rats did not express any escape behavior was recorded as the paw withdrawal threshold [9, 18–20].

### Histological and immunohistochemical assessment

The rats were euthanized by carbon dioxide on days 0 (*n* = 4), 1, 3, 5, and 7 (n = 6, Fig. 1). The whole knee joints were dissected, fixed in 4% paraformaldehyde at pH 7.4 for 7 days, decalcified in 20% EDTA solution at 4 °C for 10 days, and embedded in paraffin wax. Sagittal sections of the whole knee joint were prepared at 5 μm. Hematoxylin-eosin (HE) and Masson trichrome (MT) staining were performed as described previously [21, 22]. An Olympus BX53 microscope (Olympus, Tokyo, Japan) was used to visualize the histological sections. The % area of fibrosis was measured using ImageJ software (National Institutes of Health, Bethesda, MD, USA).

To identify the macrophages that infiltrated into the IFP, immunostaining by ED1 antibody (Abcam, Cambridge Cambridgeshire, UK) was performed, as described previously [9, 21, 23]. The number of ED1-positive cells in the IFP was counted using an Olympus BX53 microscope (Olympus, Tokyo, Japan).

### Semiquantitative evaluation of structural changes in the IFP

#### Cellularity

The perivascular region, parenchymal region of the IFP (IFP body), and superficial layer (IFP surface or synovial membrane) were chosen from a section stained with HE (representative pictures are indicated in Fig. 3). Cellularity of each section was semi-quantitatively evaluated according to the criteria described in Table 1 and representative pictures were indicated in Additional file 1: Figure S1A. Data were indicated as a mean value of 3 independent sections (day 0, *n* = 4; day 1, 3, 5, and 7 samples, *n* = 6).

**Table 1:** Semi-quantitative evaluation of structural changes in IFP: Cellularity

Score	0	1	2	3
Perivascular	Normal	Slightly increased	Moderately increased	Markedly increased
IFP body	Normal	Slightly increased	Moderately increased	Markedly increased
IFP surface	≦Single cell layer	2~3 Cell layers	4~5 Cell layers	≧ 6 Cell layers (6 or more)

#### Macrophage infiltration

Serial sagittal sections were stained with ED-1 antibody to visualize macrophages that infiltrated in IFP. The three separate areas, perivascular region, parenchymal region of IFP (IFP body), and superficial layer (IFP surface or synovial membrane), were chosen from the same section, and the number of macrophages was counted at a magnification of 200 times (representative pictures are indicated in Fig. 4). Data were indicated as a mean value of 3 independent sections (day 0, *n* = 4; day 1, 3, 5, and 7 samples, *n* = 6).

#### Fibrosis

MT staining was performed to visualize collagen fiber deposition in the IFP. The three separate areas (perivascular region, parenchymal region of IFP [IFP body], and superficial layer [IFP surface or synovial membrane]) were chosen from the same section, and % area of fibrosis was measured using ImageJ software (representative pictures are indicated in Fig. 5). Data were indicated as a mean value of 3 independent sections (day 0, n = 4; day 1, 3, 5, and 7 samples, n = 6). Semiquantitative evaluation was performed according to the criteria described in Table 2 and representative pictures were indicated in Additional file 1: Figure S1B.

**Table 2:** Semi-quantitative evaluation of structural changes in IFP: Fibrosis

Score	0	1	2	3
Perivascular	Normal, 0–20% of total area	Low, 20–40% of total area	High, 40–60% of total area	Extensive, > 60% of total area
IFP body	Normal, 0–20% of total area	Low, 20–40% of total area	High, 40–60% of total area	Extensive, > 60% of total area
IFP surface	Normal, 0–20% of total area	Low, 20–40% of total area	High, 40–60% of total area	Extensive, > 60% of total area

### Statistical analysis

**Fig. 2:**
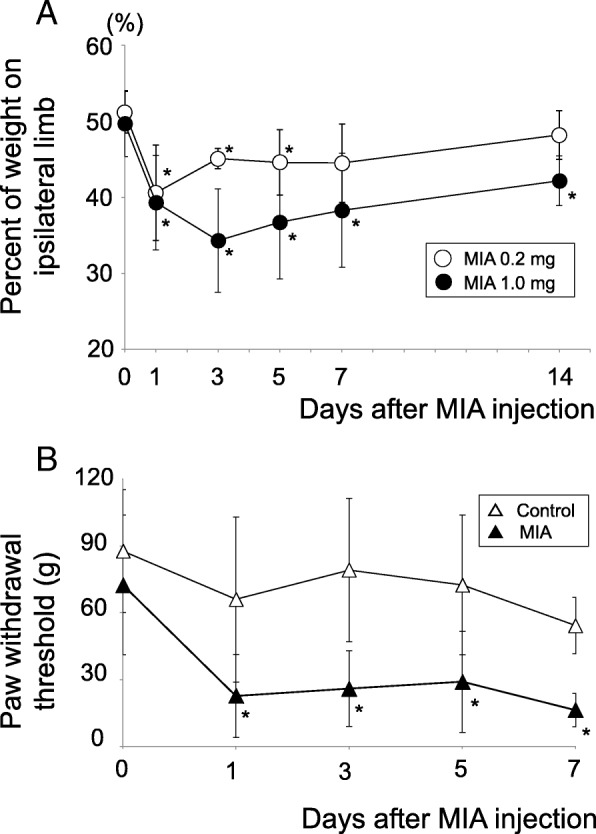
Pain avoidance behavior tests. (**a**) Incapacitance test. Weight bearing asymmetry between ipsilateral (MIA side) and contralateral (control side) limbs was measured and the percent weight of ipsilateral limb was calculated as described previously [13]. The number of samples (rats) in each time point was as follows; High-dose (1.0 mg) group, days 0, 1, 3, 5, and 7: *n* = 12, day 14: *n* = 6. Low-dose (0.2 mg) group, days 0, 1, 3, 5, and 7: *n* = 8, day 14: n = 6. Steel tests were performed to calculate if the values in each time point were statistically different from those in day 0. Open circles; 0.2 mg group. Closed circles; 1.0 mg group. Asterisks indicate the *p* value is less than 0.05. (**b**) von Frey Hair test. Time course changes of paw withdrawal threshold in high-dose group. Steel tests were performed to calculate if the values in each time point were statistically different from that in day 0. Open triangles; Contralateral side. Closed triangles; Ipsilateral side. Asterisks indicate the *p* value is less than 0.05 (*n* = 12)

**Fig. 3:**
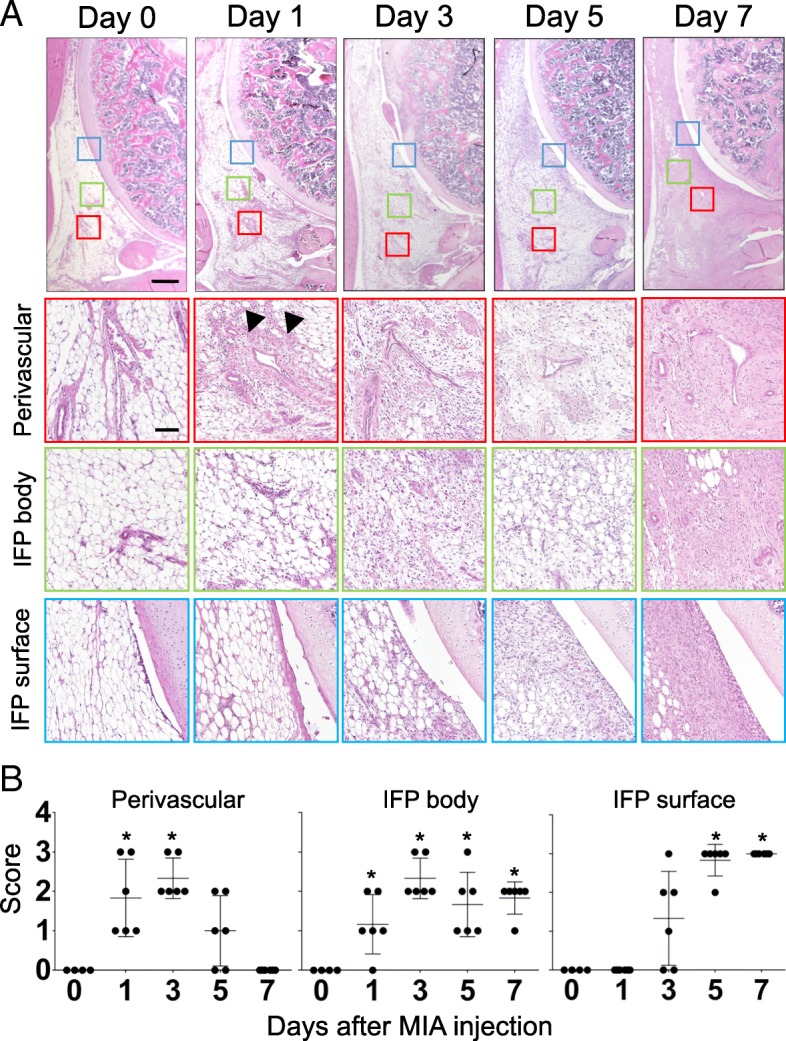
Time course histological changes of IFP (Cellularity). (**a**) Hematoxylin-eosin (HE) staining of sagittal section of the right knee joint (Top: Proximal, Left: Anterior). Boxed area in the top panels (Red: perivascular, Green: IFP body, Blue: IFP surface) are enlarged and shown below. Number of samples, day 0: *n* = 4, days 1, 3, 5, 7: *n* = 6. Scale bar, top panels: 500 μm, Second, third and bottom panels: 100 μm. Arrowheads indicate the initial hyperplastic changes in perivascular region. (**b**) Semi-quantitative evaluation of structural changes in IFP were performed according to the grading criteria described in Table 1. Three sections were randomly picked up from each sample and evaluated. Each dot indicated the mean value at each time point. Data were also indicated as mean and SD value. Number of samples in each time point, day 0: n = 4, days 1, 3, 5, 7: n = 6. Kruskal-Wallis test followed by Steel-Dwass test were performed to calculate if the difference was statistically significant. Asterisks indicated that *P* value was less than 0.05

**Fig. 4:**
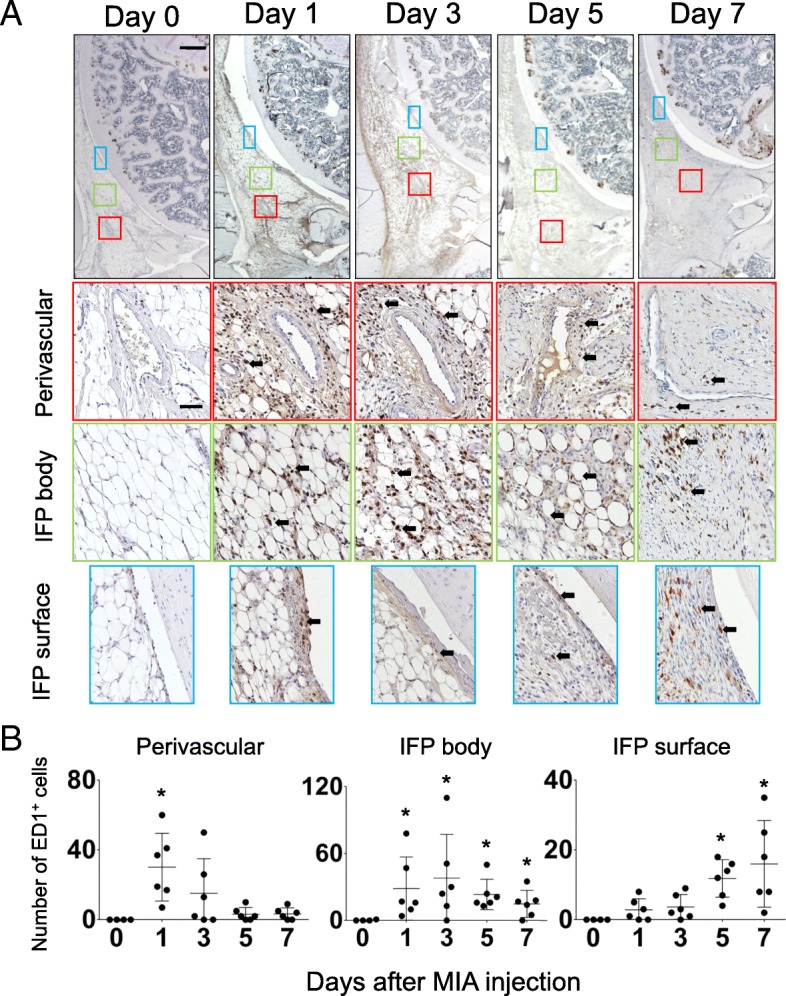
Time course changes of inflammatory cell infiltration in IFP. (**a**) Sagittal sections of the right knee joint (Top: Proximal, Left: Anterior) were stained with anti-rat ED1 antibody to identify macrophages infiltrated in IFP at each time point. Boxed area in the top panels (Red: perivascular, Green: IFP body, Blue: IFP surface) are enlarged and shown below. Number of samples, day 0: n = 4, days 1, 3, 5, 7: n = 6. Scale bar, top panels: 500 μm, Second, third and bottom panels: 50 μm. Arrows indicate examples of ED1-positive cells. (**b**) Numbers of ED1-positive macrophages in the boxed area were counted and plotted. Three sections were randomly picked up from each sample and evaluated. Each dot indicated the mean value of each time point. Data were also indicated as mean and SD value. Number of samples in each time point, day 0: n = 4, days 1, 3, 5, 7: n = 6. Kruskal-Wallis test followed by Steel-Dwass test were performed to calculate if the difference was statistically significant. Asterisks indicated that P value was less than 0.05

**Fig. 5:**
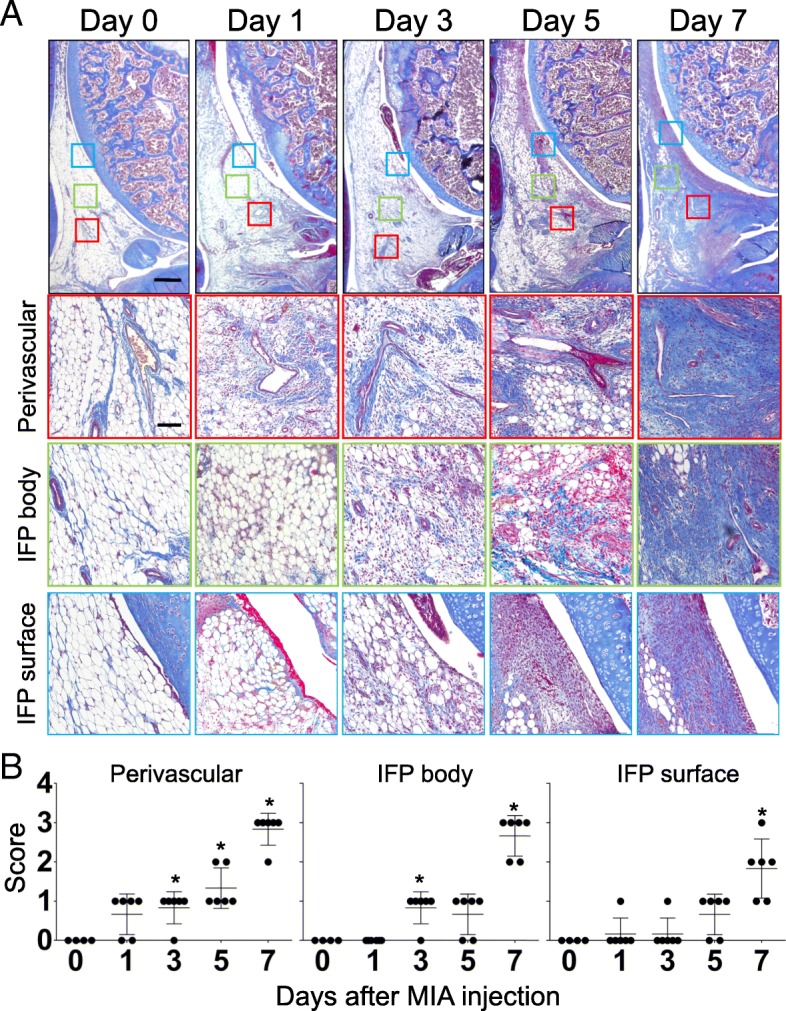
Time course histological changes of IFP (Fibrosis). (**a**) Masson Trichrome (MT) staining of sagittal section of the right knee joint (Top: Proximal, Left: Anterior). Boxed area in the top panels (Red: perivascular, Green: IFP body, Blue: IFP surface) are enlarged and shown below. Number of samples, day 0: n = 4, days 1, 3, 5, 7: n = 6. Scale bar, top panels: 500 μm, Second, third and bottom panels: 100 μm. (**b**) Semi-quantitative evaluation of structural changes in IFP were performed according to the grading criteria described in Table 2. Three sections were randomly picked up from each sample and evaluated. Each dot indicated the mean value at each time point. Data were also indicated as mean and SD value. Number of samples in each time point, day 0: n = 4, days 1, 3, 5, 7: n = 6. Kruskal-Wallis test followed by Steel-Dwass test were performed to calculate if the difference was statistically significant. Asterisks indicated that P value was less than 0.05

Steel’s test, a multiple comparison test for comparing several treatments with a control treatment, was employed to analyze the results of incapacitance test and von Frey Hair test (Fig. 2). Kruskal-Wallis test followed by Steel-Dwass test was employed to analyze the results of histological evaluations between 5 independent time points (Figs. 3, 4, and 5). All statistical analyses were performed using EZR software [24]. *P*-values < 0.05 were considered significant.

## Results

### Intra-articular injection of high-dose MIA induces persistent knee pain in rats

As described previously [13] and in this study much in detail, intra-articular injection of MIA quickly reduced weight bearing of the ipsilateral limbs on day 1 regardless of the dose (0.2 mg or 1.0 mg, Fig. 2a). In the low-dose (0.2 mg) group, this reduction continued until days 5 and then gradually alleviated to the pre-experimental levels after day 7. In contrast, weight bearing of the ipsilateral limbs reduced throughout the experimental period in the high-dose (1.0 mg) group (Fig. 2). These data suggest that some biological events that occurred between day 5 and 7 after MIA injection may play important roles during persistent pain development. Another indication, progressive tactile hypersensitivity, which contributes to a cumulative allodynia during inflammation, was also comparable with that of our previous report (Fig. 2b) [13]. These may verify the reproducibility of the pain avoidance behavior in our experimental models.

### Time course analyses of structural changes in the synovial membrane and IFP

Pain avoidance behavior tests indicated that persistent pain generation occurred on day 7 in the high-dose group. We performed histological assessments on days 0, 1, 3, 5, and 7 after MIA injection to identify biological events around the knee joint in the high-dose group (1.0 mg). Figure 3 presents the cell number changes over time in the perivascular region, IFP body (parenchymal region), and IFP surface (synovial membrane). As shown in this figure, the first sign of the structural change was observed in the perivascular region (Fig. 3a, indicated by the arrowhead). We found that the number of cell nuclei stained by hematoxylin immediately increased after MIA injection on day 1 and then reduced after day 5 in the perivascular region. Cell kinetics was semiquantitatively evaluated and plotted in Fig. 3b. Eosin staining, which visualizes both the cytoplasm and extracellular connective tissues, also indicated the surge of cellularity in the perivascular region as early as day 1 after MIA injection (Fig. 3a). Unlike that of hematoxylin, staining intensity of eosin did not decrease after day 5, suggesting the accumulation of extracellular matrices after the surge of cell numbers in the perivascular region. Cell migration kinetics in the perivascular region seemed to be followed by that in the IFP body (Fig. 3a). The number of the cells gradually increased on day 3 and was continuously high until day 7. Interestingly, staining intensity of eosin dramatically increased between days 5 and 7 without increasing the number of nuclei (stained by hematoxylin), indicating extensive extracellular matrix apposition during this period (Fig. 3a). Hyperplastic changes in the IFP surface (synovial membrane) initiated on day 3 after MIA injection and continued throughout the experimental period (Fig. 3a). These data suggest that the inflammatory response may start around the perivascular region and then move to the IFP surface despite the intra-articular injection of MIA. Representative images of the time course changes in the contralateral side (intra-articular injection of PBS) are shown in Additional file 2: Figure S2A (the top row).

Next, to analyze the changes in inflammatory cell number after intra-articular injection of MIA, immunostaining of IFP by ED-1 antibody, which identifies macrophages, was performed. As presented in Fig. 4, rapid accumulation of macrophages was observed on day 1 in the perivascular region (Fig. 4a, arrows). These macrophages spread throughout the IFP body on day 3 and reached the IFP surface on day 5 (Fig. 4a). Macrophages that infiltrated into the perivascular region and IFP body quickly disappeared after day 5 (Fig. 4a). Semiquantitative analyses also support these observations (Fig. 4b). Representative images of the time course changes in the contralateral side (intra-articular injection of PBS) are shown in Additional file 2: Figure S2 (the second row from the top).

Since HE staining suggested extensive extracellular matrix apposition between days 5 and 7 after MIA injection in the high-dose group, we examined collagen fiber accumulation and its time course changes in the IFP. As presented in Fig. 5, fibrotic changes in the IFP started from the perivascular region on day 1. Then, scattered fibrotic areas in the IFP body increased between days 3 and 5. Interestingly, fibrosis rapidly increased between days 5 and 7, and almost all the adipocytes in the parenchymal region of the IFP disappeared at day 7 (Fig. 5). Representative images of the time course changes in the contralateral side (intra-articular injection of PBS) are shown in Additional file 2: Figure S2 (the bottom row).

## Discussion

Inflammatory response plays important roles in joint pain development and its persistence. However, it is not clear whether OA-related persistent pain plays an important nociceptive role and to what degree. In this study, we described the time course structural changes in the IFP in the MIA-induced rat knee arthritis model. As described previously, the severity of joint inflammation and articular cartilage degeneration was dependent on the dose of MIA injected. In the low-dose MIA model, joint inflammation was transient, and hyperplastic changes in the IFP surface (synovial membrane) were almost completely alleviated on day 14 [12, 13]. In contrast, IFP inflammation persisted throughout the experimental period (28 days) in the high-dose MIA model [12, 13]. In this study, we showed that time course changes in pain avoidance behavior (weight bearing) had a good association with that in IFP inflammation. Weight bearing in the ipsilateral knee joint was significantly reduced as early as day 1 after MIA injection instead of the dose. Immunohistochemical analyses indicated that extensive infiltration of macrophages was observed as early as day 1. In the low-dose MIA model, this pain avoidance behavior continued on day 5 and returned to the pre-experimental levels on day 14, at the time when the hyperplastic changes in the IFP surface were almost completely alleviated. These data suggest that the major cause of acute joint pain is nociception induced by acute inflammatory response in the IFP. In contrast, weight bearing reduced throughout the experimental period in the high-dose model. Thus, we considered that some biological events, those involved in the process of persistent pain development, occur on the first 7 days after MIA injection. Based on these observations, we performed time course histological analyses and showed that extensive collagenous fiber accumulation, which causes irreversible structural changes in the parenchymal region of the IFP, occurred between days 5 and 7 after MIA injection.

**Fig. 6:**
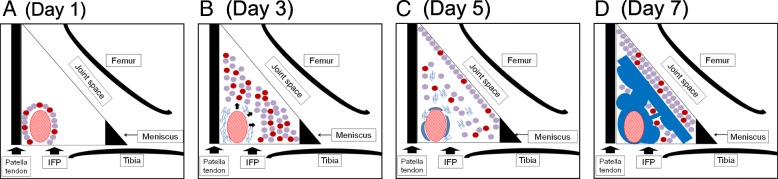
Schematic diagram of time course histological changes in IFP after MIA injection. (**a**) Day 1: Inflammatory response initiates around the perivascular region. Cellularity of the perivascular region is increased. Circles in red; inflammatory cells. Circles in purple; parenchymal cells. (**b**) Day 3: Inflammatory cell infiltration into parenchymal region of IFP. Deposition of extra-cellular matrices (indicated in blue lines) is observed in the perivascular region. (**c**) Day 5: Hyperplastic changes are observed in the IFP surface (synovial membrane). (**d**) Day 7: Extensive fibrosis (indicated in blue) is observed in the IFP

Results from cellular kinetics studies and immunohistochemical analyses are summarized in Fig. 6. The inflammatory response seemed to initiate around the perivascular region and increased cellularity in the parenchymal region followed, in which adipocytes were replaced by fibroblastic cells at 5 days (Fig. 6a, b, and c). Previous studies suggested that synovial fibroblasts function as sentinel cells that can sense tissue damage in the joint [25]. The process of tissue damage recognition by synovial fibroblasts is considered to occur through binding of DAMPs (damage-associated molecular patterns) to their receptors, such as TLR4 (Toll-like receptor 4) [25, 26]. Engagement of TLR4 activates MyD88 (myeloid differentiation primary response protein 88) signaling pathways within synovial fibroblasts which induce the release of various pro-inflammatory cytokines and chemokines [25, 27]. Although the underlying molecular pathways have not identified, we consider that these factors may initiate acute inflammatory responses including macrophage infiltration into the perivascular region at 1 day. These factors may also play important roles to amplify the inflammatory response in the parenchymal region at 3 days (Fig. 6b). We expect that IL1β and TNFα may be included in these process since these molecules have reported to enhance the proliferation of synovial fibroblasts [28, 29]. These time course changes in cell number were observed just before the occurrence of extensive fibrosis in the parenchymal region of the IFP (Fig. 6c and d). Immunohistochemical analyses indicated that infiltration of ED1-positive macrophages in the perivascular and parenchymal regions of the IFP was observed prior to the increased cellularity in each region and disappeared before extensive fibrosis occurred. These data suggest that macrophages may play important roles in both proliferation of fibroblastic cells and collagen fiber deposition in the IFP. Although the origin of fibroblastic cells that accumulated in the parenchymal region of the IFP still remained to be elucidated, we consider it feasible that residential fibroblastic cells in the IFP proliferated before extensive fibrosis occurred. However, it is still possible that fibroblastic cells could migrate from the systemic circulation through the vasculature to the IFP. Our next experimental aim is to elucidate the origin of the fibroblastic cells and roles of macrophages during the structural changes in the IFP.

The direct link between the fibrosis of IFP and joint pain persistence is not fully understood. Our previous study indicated that new calcitonin gene-related peptide-positive nerve fiber formation was observed in the fibrotic regions in the IFP. We predict that IFP fibrosis may play roles in decreasing pain threshold and increasing nociception in the joint [13].

It is reported that not only synovial inflammation but also the changes of bone marrow lesion (BML) are associated with the fluctuation of knee pain; moreover, pain resolution occurred more frequently when BMLs were smaller-sized, as reported in a human longitudinal study [30]. These data suggest that nociception in the subchondral bone may also play significant roles in establishing persistent knee pain. In this study, we focused on the inflammatory process in the IFP and did not consider the degenerative process in the articular cartilage since articular cartilage degradation did not extend to the subchondral bone, where the nociceptive receptors are located, on day 5 in our experimental models [13].

Therefore, we analyzed time course structural changes in the IFP during persistent pain development. Our data suggest that irreversible structural changes (fibrosis) were observed between days 5 and 7, which may play critical roles during persistent pain development. We predict that inhibiting the fibrotic changes in the IFP may contribute to prevention of persistent pain development in patients with OA.

## Conclusion

In this study, we showed that acute joint pain occurs along with the onset of acute inflammatory process. Irreversible structural changes in the IFP, such as extensive fibrosis, are observed prior to persistent pain development. Thus, we consider that this process may play important roles in persistent pain development.

## Additional files

Additional file 1: **Figure S1.** Semi-quantitative evaluation of structural changes in IFP. Representative images for each grade are indicated. (A) Cellularity, (B) Fibrosis. Scale bar = 100 μm. (PDF 4910 kb)

Additional file 2: **Figure S2.** Representative images of the time course changes in contralateral side (intra-articular injection of PBS). (PDF 4573 kb)

## Abbreviations

CGRP: Calcitonin gene-related peptide
EDTA: Ethylenediaminetetraacetic acid
HE: Hematoxylin and eosin
IFP: Infrapatellar fat pad
MIA: Monoiodo-acetic acid
OA: Osteoarthritis
OARSI: Osteoarthritis Research Society International
PBS: Phosphate buffered saline
PFA: Paraformaldehyde
