# Supplementary figures and images for: Time course analyses of structural changes in the infrapatellar fat pad and synovial membrane during inflammation-induced persistent pain development in rat knee joint
Source: BMC Musculoskelet Disord. 2019 Jan 5;20:8. doi: 10.1186/s12891-018-2391-1 (PMC6320593; doi:10.1186/s12891-018-2391-1)

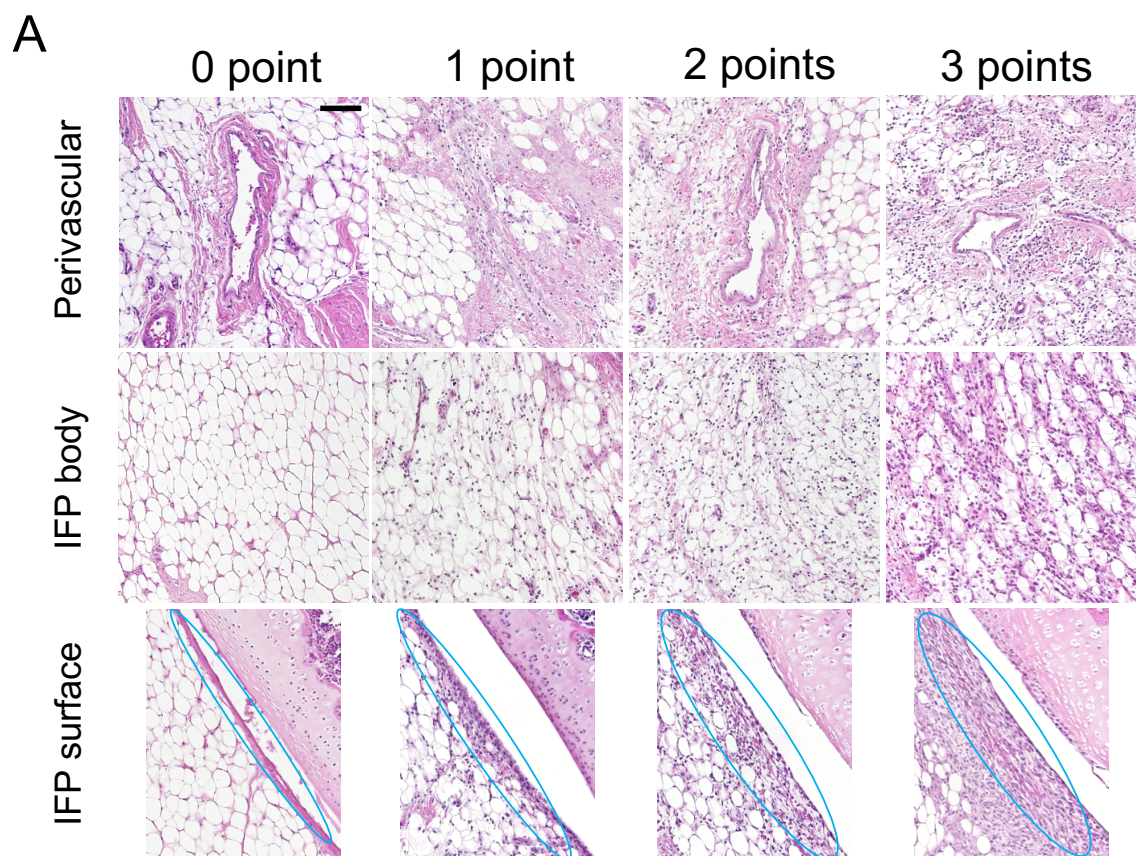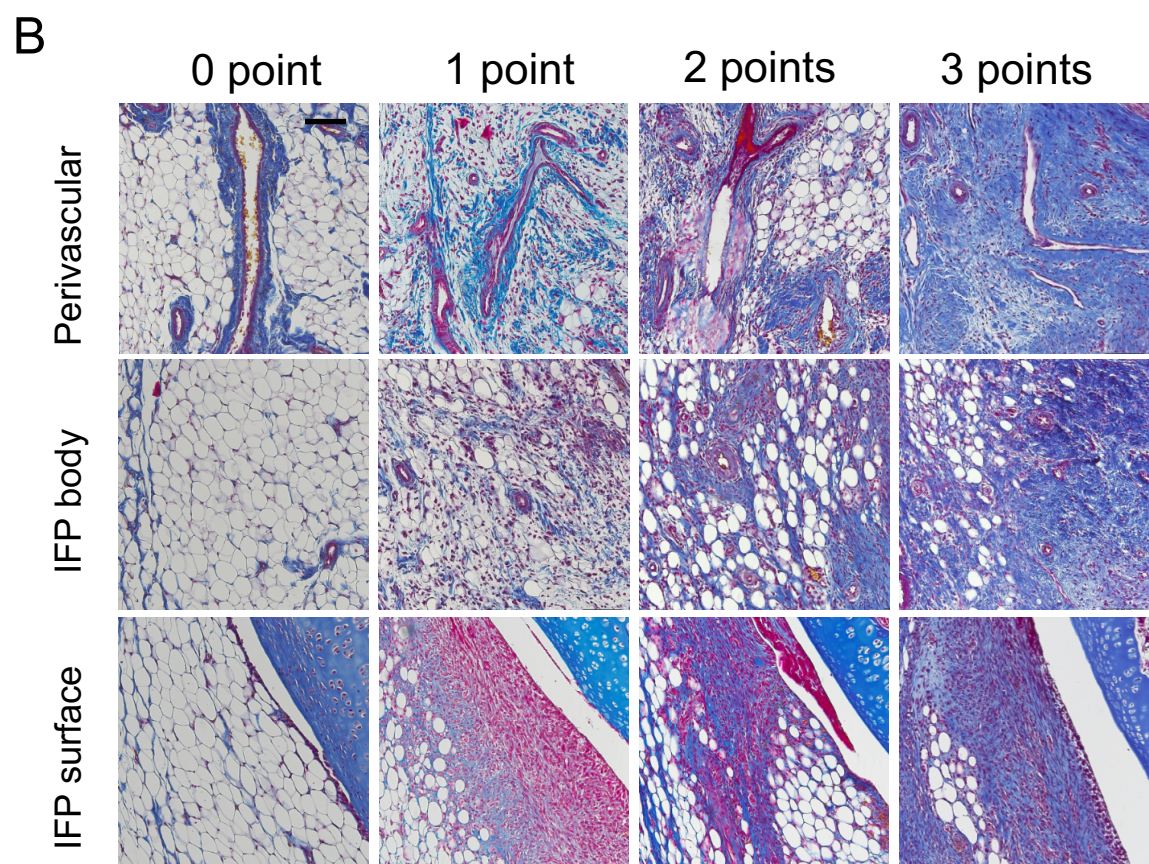

Supplement: Supplementary file 1 — Figure S1. Semi-quantitative evaluation of structural changes in IFP. Representative images for each grade are indicated. (A) Cellularity, (B) Fibrosis. Scale bar = 100 μm. (PDF 4910 kb) [file 12891_2018_2391_MOESM1_ESM.pdf]

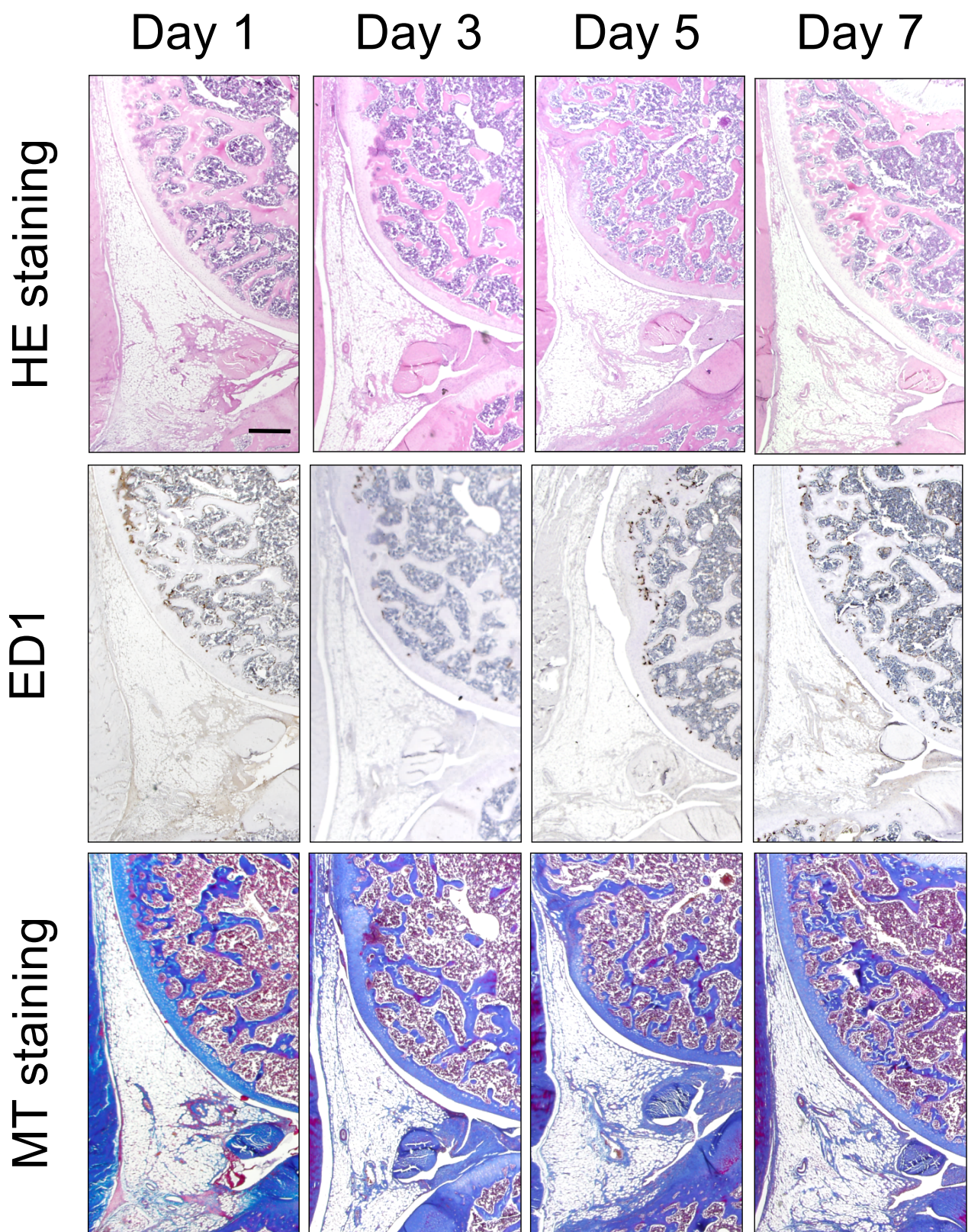

Inomata et. al., supplemental Figure 2

Supplement: Supplementary file 2 — Figure S2. Representative images of the time course changes in contralateral side (intra-articular injection of PBS). (PDF 4573 kb) [file 12891_2018_2391_MOESM2_ESM.pdf]
